# Physical long-term conditions and the effectiveness of England’s NHS Talking Therapies programme for working-age adults: findings from a South London borough

**DOI:** 10.1136/bmjment-2025-301632

**Published:** 2025-05-19

**Authors:** Amy Ronaldson, Matthew Broadbent, Brendon Stubbs, Lisa Harber-Aschan, Nicusor Sima, David Armstrong, Ioannis Bakolis, Stephani Hatch, Matthew Hotopf, Alex Dregan

**Affiliations:** 1 Department of Biostatistics and Health Informatics, King’s College London, London, UK; 2 NIHR Maudsley Biomedical Research Centre, SLAM, London, UK; 3 Department of Psychological Medicine, King’s College London, Institute of Psychiatry, Psychology, and Neurosciences, London, UK; 4 Physiotherapy, South London and Maudsley NHS Foundation Trust, London, UK; 5 Department of Sociology, Stockholm University, Stockholm, Sweden; 6 Department of Technological Sciences, University of Agricultural Sciences and Veterinary Medicine Cluj-Napoca, Cluj-Napoca, Romania; 7 Population Health Sciences, King’s College London, London, UK; 8 Biostatistics and Health Informatics, King’s College London School of Social Science and Public Policy, London, UK; 9 South London and Maudsley NHS Foundation Trust, London, UK

**Keywords:** Anxiety disorders, Depression & mood disorders, Adult psychiatry, Depression

## Abstract

**Objective:**

To assess the effectiveness of NHS Talking Therapies (NHSTT) service for working-age adults with mild to moderate depression or anxiety and to evaluate the impact of multiple physical long-term conditions (LTCs) on treatment outcomes.

**Method:**

We have linked routinely collected data from the NHSTT services in South London (UK) with primary care data for aged 18–64 years who had accessed the services between August 2008 and March 2021. The main outcome measures were NHSTT service key performance indicators of ‘recovery’ and ‘reliable improvement’. Multiple and specific physical LTCs represented the exposure of interest. Cox proportional hazard models were used to assess associations between physical LTC exposures and outcomes.

**Findings:**

Among 35 814 adults (mean age=37, 67% women) attending the NHSTT, physical LTCs were associated with moderately lower ‘recovery’ rate (adjusted HR (aHR)=0.91, 95% CI 0.88 to 0.95) relative to no LTCs. A dose–response relationship was also observed: the likelihood of ‘recovery’ decreased with the number of physical LTCs (one condition: aHR=0.95, 95% CI 0.91 to 0.98; two conditions: aHR=0.88, 95% CI 0.83 to 0.93; three conditions: aHR=0.82, 95% CI 0.75 to 0.91; four or more conditions: aHR=0.72, 95% CI 0.61 to 0.85).

**Conclusion:**

Among working-age adults, the effectiveness of NHSTT services varied with the number and type of physical LTCs. These findings highlight the need for tailored interventions for patients with multiple physical LTCs to improve treatment outcomes.

**WHAT IS ALREADY KNOWN ON THIS TOPIC**

- Treatment outcomes of NHS Talking Therapies (NHSTT) for patients with long-term conditions (LTCs) are considered to be poorer relative to those without physical LTCs.

**WHAT THIS STUDY ADDS**

- There was substantial variability in key performance indicators for NHSTT services across specific physical LTCs.
- A dose–response association was observed with the likelihood of achieving ‘recovery’ and ‘reliable improvement’ declining with the number of physical LTCs.

**HOW THIS STUDY MIGHT AFFECT RESEARCH, PRACTICE OR POLICY**

- The effectiveness of NHSTT is severely impaired when working-age adults with depression or anxiety present with multiple physical LTCs.
- NHSTT services should tailor interventions for ethnically diverse adults with specific physical LTCs.

## Background

 In 2008, the NHS Talking Therapies (NHSTT) service was established in England to provide brief psychological therapies to people with mild to moderate depression and anxiety disorders within the NHS.^1^ These services offer several brief treatments such as cognitive behaviour therapies, counselling, self-help guide, interpersonal therapy (IPT) and other, structured and manualised brief treatments. NHSTT-defined patient outcomes include ‘recovery’ and ‘reliable improvement’, which are calculated based on changes in depression and anxiety symptoms from baseline to end of treatment sessions. The recent data show that 52.2% of referred patients were deemed to have achieved ‘recovery’ by the end of treatment, with over two-thirds (68.2%) showing ‘reliable improvement’.^2^ Although this meets the government target of 50% of referrals moving to ‘recovery’, there is significant variability in treatment response.^3^

In 2011, the UK government published ‘No Health without Mental Health’, a cross-governmental mental health outcomes strategy, highlighting the need for NHSTT services to accommodate patients with physical long-term conditions (LTCs).^4^ A recent evaluation of NHSTT found poorer outcomes compared with patients without reported LTCs after controlling for sociodemographic and clinical baseline variables.^3 5^ Evidence is more positive with regard to employment and reduced healthcare utilisation,^6^ although this evidence comes from a restricted number of LTCs, such as cardiovascular disease, diabetes and chronic obstructive pulmonary disease (COPD). The consequence of an LTC, can vary considerably with some (eg, COPD) posing serious health implications, while others (eg, hypertension, obesity), may be more manageable.

The LTC pathway within the NHSTT (NHS TT for depression and anxiety) offers a more LTC-tailored approach aiming to integrate mental health interventions with management of coexisting LTC. Official evidence suggests that such personalised approaches may enhance patient engagement and adherence to therapies, while leading to improvement in both mental and physical health outcomes.^7^ However, there is limited evidence-based knowledge about the effectiveness of NHS TTad across specific LTCs or indeed patients living with multiple LTCs (MLTCs). The latter is of key public health relevance given that approximately 27% of UK adults in primary care live with MLTC^8^ and this is expected to rise considerably in the coming years.^9^ Living with MLTC is associated with increased risk of depression and anxiety,^10^ challenging the provision of optimal care.^11^

The present study used linked primary care and NHSTT data to examine the extent to which LTCs affected outcome rates in a large sample of working-age adults (18–64 years). Our study focused on working-age adults, the intended population target for the initial NHSTT in LTC programme. While MLTC research commonly focuses on older adults, there is limited evidence on the effectiveness of the NHSTT programme in younger populations with predominantly mental health conditions.^10^ This group presents, thus, distinctive therapeutic challenges compared with older adults. We have also identified the most commonly occurring physical LTCs in this population and estimated service characteristics with the greatest impact on key performance indicators.

## Method

### Sample and study design

A prospective observational cohort study design was employed using linked electronic health records (EHR) data from NHSTT services and primary care records from the Lambeth DataNet (LDN). Data linkage was facilitated by the South London and Maudsley (SLaM) Clinical Record Interactive Search (CRIS) system. LDN is a pseudonymised dataset consisting of coded data (Read and SNOMED-CT medication codes) that comprises clinical, therapeutic and referral data from general practices in Lambeth, which was set up as a local resource to allow for the assessment of local health inequalities, particularly relating to ethnicity and social deprivation.

The primary sample consisted of 47 097 working-age (18–64 years) adults who were registered with a general practitioner (GP) in Lambeth and who had accessed Lambeth NHSTT services between August 2008 (when the first NHSTT appointment was recorded) and March 2021 (when the data were linked and extracted). The focus on working-age adults was motivated by the fact that this is the group with the highest absolute number of MLTC and those targeted for the original NHSTT service. In line with previous research examining NHSTT outcomes,^12^ patients with symptoms of anxiety or depression who had a course of NHSTT treatment (two or more treatment sessions) and who were defined as ‘cases’ at the start of treatment (ie, they had symptoms of anxiety and/or depression that met the threshold for ‘caseness’—see the outcomes section for more detail) comprised the sample for the current study (n=35 814). A patient flow diagram is provided in online supplemental figure S1.

### Exposure measurements

#### Long-term conditions

Information on pre-NHSTT physical LTCs was derived primarily from clinical diagnoses recorded in primary care records up to the date of the first treatment session in the NHSTT services. In addition, we have used self-reported data on LTCs recorded at the time of the NHSTT assessment to complement primary care data. The combination of the different data sources ensured comprehensive data on LTCs across the care continuum. Based on previous work and data availability,^13^ a total of 31 physical LTCs were included in the analysis (online supplemental table S1). The conditions overlap with those proposed in a recent systematic review to be included in the definition of MLTCs.^14^ For analysis purposes, we have estimated the impact of specific LTCs (eg, cancer, diabetes, hypertension, psoriasis) on NHSTT treatment outcomes. To reflect the evidence that patients with depression and anxiety often live with MLTCs,^15^ we have also created a variable that used two or more coexisting physical LTCs as the threshold for classifying patients into MLTC (vs none or one LTC).^13^ The definition of MLTC included the diagnosis of two or more distinctive LTCs recorded at any given timepoint between 2008 and 2021.

### Clinical and NHSTT service characteristics among those with ≥1 LTC

To explain potential variability in NHSTT treatment outcomes for patients with above LTCs, we have considered both clinical and service-specific attributes. Clinical characteristics included baseline Patient Health Questionnaire-9 (PHQ-9) and General Anxiety Disorder-7 (GAD-7) scores taken at the first treatment session as indicators of symptom severity. NHSTT service characteristics included number of sessions, the session ‘did not attend’ rate (when patients do not attend a scheduled therapy session without prior notice), referral to treatment time (the period from date of referral to the date of first treatment appointment in days), referral source and the type of treatment intensity received. Three categories of NHSTT service referral source were considered: GP/primary care, self-referral and other (secondary care (psychiatric or physical services), community services). NHSTT services follow a stepped care model in line with National Institute for Health and Care Excellence guidelines for the management of depression and anxiety disorders,^16^ offering the least intrusive, most efficient therapy initially, and stepping up to more intensive therapy as required.^1^ The categories of treatment intensity considered in the study were step 2 (eg, guided self-help, digital digital cognitive-behavioural therapy (CBT), psychoeducational groups), step 3 (individual CBT, IPT, eye movement and desensitisation and reprocessing) and other (triage, therapies where the intensity was not recorded).

### Outcome measures

The current study used two NHSTT-defined key performance indicators to evaluate the effectiveness of the programme in patients with LTCs. First, *time to recovery* rate defined as the proportion of patients that score below the clinical threshold on standardised measures of depression and anxiety (eg, PHQ-9 and GAD-7) at the end of treatment sessions.^12^ Second, *time to reliable improvement* measure assessing the proportion of patients where scores on the depression and/or the relevant anxiety measure have reduced by a reliable amount and neither measure has shown a reliable increase.

The PHQ-9^17^ and the GAD-7 assessments^18^ are used in NHSTT services to determine the effectiveness of the programme (ie, ‘recovery’ and ‘reliable improvement’) relating to depression and anxiety. Scores of 10 or more on the PHQ-9 indicate caseness for depression and a reduction of 6 or more points indicates ‘reliable improvement’. Scores of 8 or more on the GAD-7 indicate caseness for anxiety and a reduction of 4 or more points indicates ‘reliable improvement’.

### Covariates

To increase the validity and accuracy of the findings, we have adjusted for various variables that are potential confounders for effectiveness of NHSTT for patients with LTCs. These confounders included demographic (age, gender, ethnicity), socioeconomic (employment status, social deprivation) and baseline symptom severity. Age reflected the age at which patients attended their first NHSTT session and was included as a continuous variable. Detailed information about ethnicity was gathered in NHSTT services. In the current study, we included seven ethnic groups: white, Black Caribbean, Black African, Black Other, Asian, mixed ethnicity, other ethnicity. We also included a separate category of patients whose ethnicity was unknown (not stated). A detailed description of how the ethnic groups were derived is provided in online supplemental table S2. Employment status was based on self-reports and classified patients into employed, unemployed, retired, student, disabled and homemaker/carer. Social deprivation data were obtained from primary care and were assessed by mapping patient postcodes to the lower super output area and assigning the index of multiple deprivation (IMD—2015) score for that area. IMD provides a composite score for a range of neighbourhood-level indicators of deprivation including income, employment, education, health and crime. Scores were grouped into quintiles 1–5 with ‘1’ corresponding with least deprived and ‘5’ with most deprived. Patients’ scores on PHQ-9 and GAD-7 instruments at the first assessment were used as indicators of baseline severity symptoms for depression and anxiety, respectively.

### Statistical analysis

Descriptive statistics were used to describe differences in baseline characteristics among the LTC and non-LTC groups. Variables were summarised as means and SD and percentages. Comparisons across different sociodemographic and treatment-related factors between those with and without physical LTCs at the initial assessment were performed using Mann-Whitney U and χ^2^ tests.

Cox proportional hazard regression models were fitted to estimate the association between specific physical LTCs and MLTC with NHSTT treatment outcomes. Cox regression was preferred over logistic regression analysis as it allows to model both the occurrence and timing of the event. Unlike logistic regression that models reliable recovery at a fixed time point, Cox regression enables the modelling of variability in treatment duration and censoring (eg, some patients might drop out after the second or third session). In this respect, Cox regression allows for a more nuanced understanding of trajectories towards recovery and improvement within the NHSTT. Cox regression was also used to model associations between patient clinical characteristics and service attributes and outcomes among those with pre-existing physical LTCs. These analyses included ‘time to recovery’ and ‘time to reliable improvement’ measures as the outcomes and LTC status and baseline covariates as predictor variables. Patients were followed up from the initial assessment (start date) until the date of the final treatment session (end date). The timescale was calendar time (days). We have considered the latest NHSTT referral episode in analyses, to reflect current service effectiveness. All analyses controlled for *a priori* confounders including age, gender, ethnicity, baseline depressive and anxiety symptoms, and social deprivation. The proportionality assumption was tested using Schoenfeld residuals and found not to be violated. Data were missing for social deprivation (3%), employment status (2%) and for treatment intensity (2%). Multiple imputation by chained equations with 10 imputations was performed to deal with missing data bias.^19^ Multiple imputation included all study variables (covariates, exposures and outcomes) to account for their complex inter-relationships.

### Additional analyses

As the final sample comprised those who had attended at least two NHSTT sessions and who met definitions for caseness for depression and/or anxiety, we performed a sensitivity analysis comparing the final sample with the excluded sample. Also, in the small sample of patients (N=6408) with available data on functional improvement, we have tested for a dose–response relationship with the number of LTCs. The functional improvement assessment is measured via the Work and Social Adjustment Scale—a self-reported questionnaire used to assess the impact of therapy on patients’ daily functioning (eg, work, social and private activities, personal relationships, home management). Thus, time to functional improvement enables a more holistic view of NHSTT outcomes, beyond symptoms reduction. All analyses were conducted in STATA V.17.0 (Stata Corp LLP, College Station, Texas).

## Results

### Sample characteristics

The final sample comprised 35 814 working-age adults who had accessed NHSTT services before March 2021. All participants attended at least two sessions and had clinical ‘caseness’ for depression and/or anxiety at the first NHSTT assessment.

Table 1 illustrates the sample characteristics by LTC status. Overall, 17 739 (50%) of participants had at least one physical LTC before accessing the service. Those with ≥1 LTC were slightly older (mean age: 37 vs 33 years) than those with no LTCs and were more likely to be from an ethnic minority group (38% vs 33%). Patients with one or more LTCs were also more likely to be unemployed (25% vs 20%) and to be within the most deprived IMD quintile (21% vs 19%).

**Table 1:** Sample characteristics for those with and without pre- NHSTT physical LTCs

	≥1 physical LTC(n=17 739, 49.5%)	No physical LTC(n=18 075, 50.5%)	P value
	M±SD, N (%)	M±SD, N (%)	
Age			**<0.001**
18–24 years	3510 (20)	4248 (24)	
25–35 years	6274 (35)	8362 (46)	
36–45 years	3598 (20)	3424 (19)	
46–55 years	2960 (17)	1633 (9)	
56–65 years	1397 (8)	408 (2)	
Female	11 761 (66)	11 880 (66)	0.205
Ethnicity			**<0.001**
White	11 008 (62)	12 114 (67)	
Black Caribbean	2234 (13)	1491 (8)	
Black African	754 (4)	798 (4)	
Black Other	526 (3)	344 (2)	
Asian	808 (5)	845 (5)	
Mixed ethnicity	1291 (7)	1184 (7)	
Other ethnicity	562 (3)	785 (4)	
Missing	556 (3)	514 (3)	
Employment status			**<0.001**
Employed	11 429 (64)	1281 (71)	
Unemployed	4354 (24)	3738 (20)	
Full-time student	829 (5)	1064 (6)	
Retired	140 (1)	36 (0)	
Homemaker/carer	355 (2)	251 (1)	
Long-term sick/disabled	456 (3)	213 (1)	
Missing	176 (1)	190 (1)	
IMD			**<0.001**
1 (least deprived)	3482 (20)	3759 (21)	
2	3357 (19)	3791 (21)	
3	3405 (19)	3695 (20)	
4	3704 (21)	2459 (19)	
5 (most deprived)	3791 (21)	3371 (19)	
Number physical LTCs (median, IQR)	1 (1 to 2)	–	**<0.001**
Number of NHSTT sessions (median, IQR)	8 (5 to 12)	8 (5 to 11)	**0.004**
Session DNA rate (median, IQR)	0 (0 to 1)	0 (0 to 1)	0.072
Duration of first NHSTT episode (weeks) (median, IQR)	58 (5 to 131)	56 (2 to 122)	**<0.001**
NHSTT referral source			**<0.001**
GP	3435 (19)	3506 (19)	
Self-referral	12 724 (72)	13 220 (73)	
Other*	1580 (9)	1349 (8)	
RTT (days) (median, IQR)	9 (3 to 22)	8 (3 to 21)	**<0.001**
NHSTT intervention			**<0.001**
Step 2	6209 (35)	6689 (37)	
Step 3	7096 (40)	6869 (38)	
Other†	4434 (24)	4518 (25)	
Total number of NHSTT episodes	1.36±0.75	1.27±0.63	**<0.001**
Baseline PHQ-9	15.83±5.61	15.35±5.57	**<0.001**
Baseline GAD-7	14.32±4.34	14.16±4.27	**<0.001**
‘Recovery’	6554 (37)	7050 (39)	**<0.001**
‘Reliable improvement’	6413 (36)	6630 (37)	0.299

* Other referral sources include general hospital/psychiatric services, community services, other.

† Step 2: low-intensity interventions, for example, guided self-help, behavioural activation, psychoeducation and computerised CBT; step 3: high-intensity interventions, for example, face-to-face CBT, interpersonal therapy, behavioural activation, counselling, psychodynamic therapy; other: triage and therapies where the intensity was not recorded.

DNA, did not attend; GAD-7, General Anxiety Disorder-7; GP, general practitioner; IMD, index of multiple deprivation; LTC, long-term condition; M, mean; NHSTT, NHS Talking Therapies; PHQ-9, Patient Health Questionnaire-9; RTT, referral to treatment time.

Depression and anxiety symptom severity were higher in those with ≥1 LTC, and they were more likely to receive a high-intensity (step 3) intervention (41% vs 38%). In terms of outcomes, patients with ≥1 physical LTC showed lower likelihood of achieving ‘recovery’ after the first episode of care (37% vs 39%), but there was no significant difference in terms of likelihood of achieving ‘reliable improvement’.

### MLTCs and treatment outcomes

**Figure 1:**
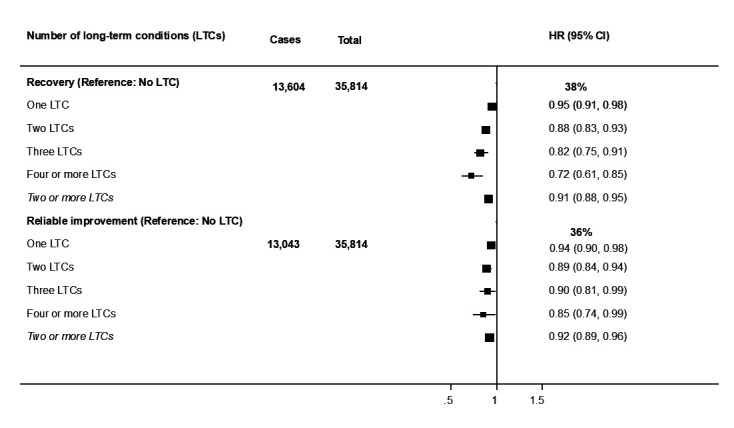
Forest plot showing the adjusted association between number of long-term conditions and NHSTT treatment outcomes. The analyses adjusted for all study covariates (age, gender, ethnicity, employment status, index of multiple deprivation, baseline symptom severity). NHSTT, NHS Talking Therapies.

Associations between pre-NHSTT physical LTCs and NHSTT outcomes are presented in figure 1. In fully adjusted models, patients with ≥1 physical LTCs showed lower likelihood of achieving ‘recovery’ after their first NHSTT episode (adjusted HR (aHR)=0.91, 95% CI 0.88 to 0.95) relative to those without an LTC. Notably, a dose–response relationship was evident with the likelihood of achieving ‘recovery’ declining as the number of LTCs increased (one condition: aHR=0.95, 95% CI 0.91 to 0.98; two conditions: aHR=0.88, 95% CI 0.83 to 0.93; three conditions: aHR=0.82, 95% CI 0.75 to 0.91; four or more conditions: aHR=0.72, 95% CI 0.61 to 0.85).

After adjusting for relevant covariates, patients with ≥2 physical LTCs had lower likelihood of achieving ‘reliable improvement’ (aHR=0.92, 95% CI 0.89 to 0.96) compared with their counterparts without LTCs. Similar to the ‘time to recovery’ analysis, the likelihood of achieving ‘reliable improvement’ declined with the number of LTCs from 0.94 (95% CI 0.90 to 0.98) among patients with one condition to 0.85 (95% CI 0.74 to 0.99) among those with four or more LTCs. The proportion of patients at likelihood of ‘recovery’ and likelihood of achieving ‘reliable improvement’ according to the number of physical LTCs is presented in online supplemental figure S2, alongside the average number of treatment sessions attended during the first episode of care.

### Specific LTCs and treatment outcomes

Among those with ≥1 physical LTC, significant associations between the most prevalent (>5%) conditions and NHSTT outcomes are presented in table 2. The most prevalent conditions were psoriasis/eczema (45%), asthma (33%), irritable bowel syndrome (IBS) (16%), hypertension (11%), hearing problems (9%), diabetes (8%) and thyroid problems (6%) (online supplemental table S3). The findings revealed that having asthma, IBS, hypertension or diabetes was associated with a reduced likelihood of achieving ‘recovery’. IBS and diabetes conditions were also associated with reduced likelihood of achieving ‘reliable improvement’.

**Table 2:** Associations between the most prevalent physical long-term conditions and treatment outcomes in patients with at least one pre- NHSTT physical LTC (N=17 739)

	Recovery		Reliable improvement	
	HR (95% CI)	P value	HR (95% CI)	P value
Specific long-term conditions				
Psoriasis/eczema	**1.08 (1.03 to 1.14**)	**0.001**	1.02 (0.97 to 1.07)	0.477
Asthma	**0.93 (0.89 to 0.98**)	**0.012**	1.01 (0.96 to 1.06)	0.753
IBS	**0.90 (0.84 to 0.96**)	**0.001**	**0.85 (0.79 to 0.91**)	**<0.001**
Hypertension	**0.91 (0.83 to 0.99**)	**0.037**	0.98 (0.90 to 1.07)	0.664
Hearing problems	0.95 (0.88 to 1.04)	0.302	0.95 (0.87 to 1.03)	0.232
Diabetes	**0.83 (0.75 to 0.92**)	**<0.001**	**0.90 (0.82 to 1.00**)	**0.043**
Thyroid problems	1.00 (0.90 to 1.12)	0.965	1.02 (0.91 to 1.14)	0.741

Covariates=age, gender, ethnicity, employment status, index of multiple deprivation, baseline symptoms severity. Significant associations are presented in bold.

IBS, irritable bowel syndrome; LTCs, long-term conditions; NHSTT, NHS Talking Therapies.

### NHSTT service characteristics and treatment outcomes

The impact of clinical and service characteristics on NHSTT outcomes among those with ≥1 physical LTCs is presented in table 3. The findings revealed that self-referrals were associated with higher likelihood of achieving both ‘recovery’ (aHR=1.15, 95% CI 1.07 to 1.23) and achieving ‘reliable improvement’ (aHR=1.10, 95% CI 1.04 to 1.18) while the opposite was observed for more intensive treatment and higher number of therapy sessions.

**Table 3:** Service attributes impacting the likelihood of achieving ‘recovery’, ‘reliable improvement’, after the first episode of NHSTT in patients with ≥1 physical LTCs (N=17 739)

	Recovery		Reliable improvement	
	HR (95% CI)	P value	HR (95% CI)	P value
Number of therapy sessions	0.91 (0.90 to 0.91)	**<0.001**	0.90 (0.90 to 0.91)	**<0.001**
Session DNA rate	0.96 (0.93 to 0.99)	**0.012**	0.99 (0.96 to 1.02)	0.666
Referral to treatment time (days)	1.00 (1.00 to 1.00)	0.260	1.00 (1.00 to 1.00)	0.136
NHSTT referral source				
GP	Ref		Ref	
Self-referral	1.15 (1.07 to 1.23)	**<0.001**	1.10 (1.03 to 1.18)	**0.004**
Other	0.97 (0.86 to 1.08)	0.542	0.96 (0.87 to 1.07)	0.509
NHSTT treatment intensity				
Step 2	Ref		Ref	
Step 3	0.56 (0.53 to 0.59)	**<0.001**	0.58 (0.55 to 0.61)	**<0.001**
Other	0.54 (0.44 to 0.66)	**<0.001**	0.62 (0.51 to 0.75)	**<0.001**

Covariates=age, gender, ethnicity, employment status, index of multiple deprivation, baseline symptoms severity.

DNA, did not attend; GP, general practitioner; LTCs, long-term conditions; NHSTT, NHS Talking Therapies.

### Additional analysis

Differences in sample characteristics for the analytical sample (n=35 814) and the excluded sample (n=11 283) are presented in online supplemental table S4. The findings for time to functional improvement (online supplemental figure S3) revealed that patients with two or more coexisting LTCs experienced lower likelihood of achieving functional improvement relative to those with none or one LTC (aHR=0.87, 95% CI 0.79 to 0.95). No clear dose–response relationship emerged, probably due to insufficient statistical power (ie, wide CIs for three and four or more LTC categories).

## Discussion

In a prospective cohort of working-age adults from an ethnically and socioeconomically diverse area of London, pre-existing LTCs were associated with reduced effectiveness of brief talking therapies. Specifically, the likelihood of achieving ‘recovery’ (38%) and/or achieving ‘reliable improvement’ (36%) rates in our sample were lower compared with published official figures (45%) and the expected NHS target set for ‘recovery’ rate (50%).^2^ The lower than expected likelihood of achieving ‘recovery’ rate observed here may be due to higher rates of MLTC in the study population, which further complicates the therapeutic process. This suggestion is supported by the observation that the likelihood of achieving ‘recovery’ and ‘reliable improvement’ rates tended to decline in a dose–response fashion, as the number of LTCs increased. A similar picture emerged with respect to time to functional improvement measure in a subsample of patients with available information. The small sample size for the latter impedes any robust conclusion, however. Given the critical role of functional status in people’s daily lives, there should be greater efforts to improve its recording (eg, patient education, integration within EHR, use of mobile apps/online tools) in the NHSTT service.

While the service now extends to patients with specific LTCs, such as diabetes, COPD and IBS, our results suggest that NHSTT is less optimal for these patients. Conversely, the presence of skin conditions, such as eczema and psoriasis, was associated with enhanced likelihood of achieving ‘recovery’. One possible explanation for the variation in treatment outcomes for NHSTT by specific conditions includes differences in demographics and treatment response. Our study population included higher proportion of women, who tend to report less severe psoriasis symptoms and respond better to treatment than men. Notably, findings from cluster analysis indicated that patients with combined psoriasis/eczema and IBS conditions had likelihood of achieving ‘recovery’ and/or achieve ‘reliable improvement’. These findings underscore the importance for tailored interventions that account for the specific needs of patients with complex mental and physical LTCs. Specialised targeted training for NHSTT therapists and optimising integrated care for patients with complex needs are promising avenues to be explored in future studies. Notably, our findings also revealed suboptimal treatment outcomes in ethnic minority patients in line with previous studies,^20^ supporting the need for future evaluations and continuous service improvement.

Our study findings align with previous studies in similar populations. Saunders *et al*^12^ found that 40% of working-age patients with MLTCs experienced ‘reliable recovery’, which is marginally higher than our study findings on likelihood of achieving ‘recovery’ (35%). They also found higher rates for ‘reliable improvement’ compared with our findings (66% vs 36% in our study), which might relate to sampling differences or the nature of the NHSTT service (number of sessions, treatment intensity). Our population included a higher rate of ethnic minority adults, who presented with poorer treatment outcomes. Griffiths and Griffiths^21^ with a larger sample size observed a 30%–34% recovery associated with the NHSTT, which is closer to our study estimates. Seaton *et al*^3^ found slightly lower ‘recovery’ rates (32% vs 38%) than our study, despite employing the same base sampling population. Differences in the study period and sample size (6600 in Seaton *et al* vs 35 800 in current study) may explain these differences.

It is well established that common mental health disorders often coexist with physical LTCs.^10 15 22^ Evidence suggests that NHSTT-referred patients with LTCs have higher levels of post-treatment psychological distress than those without LTCs.^5^ The dose–response relationship between the number of LTCs and likelihood of achieving ‘recovery’ rates in our study suggests that the effectiveness of NHSTT service is suboptimal for patients living with coexisting mental and physical LTCs. Several factors may account for this finding, including competing priorities for disease management in the presence of multiple coexisting disorders. For instance, the uptake or engagement with the NHSTT in patients with severe COPD may depend on acute shortness of breath or lung function symptoms. It is worth considering that the lower rates for likelihood of achieving ‘recovery’ and ‘reliable improvement’ observed in this study may also reflect poor management or deterioration in the underlying LTC, which in turn may complicate the therapy outcome. Studies that link NHSTT data with primary care data would allow superior insights into factors that may mediate the effectiveness of NHSTT programme in patients with LTCs.

Interpretation of the suboptimal treatment outcomes observed in our study should be considered in the context of ongoing NHSTT transformation during the study period. For instance, the implementation of the NHSTT in LTC pathway and targeted efforts to improve access and outcomes for ethnic minority populations were at varying stages and uptake.^23^ It is possible, thus, that the full impact of these interventions may not have been observable within the study timeframe. Also, service-level changes and staff training to support these developments likely varied across sites potentially contributing to inconsistencies in treatment delivery and outcomes.

Our study has several strengths and limitations. This study is one of the largest prospective studies with an ethnically diverse sample to evaluate the effectiveness of NHSTT services in MLTCs. The linked primary care data with accurate definition of LTCs and NHSTT service data with detailed talking therapy information enabled more robust estimates. Several limitations also need acknowledgement. The observational study design precludes causal inferences between physical LTCs and key service outcomes. While we accounted for key covariates, we cannot exclude the possibility of residual confounding, such as coprescribing and patient or care system attributes (eg, discrimination, stigma).^23^ Another limitation of our study was the lack of information on whether the provision of therapy was adopted in response to patients’ disclosure of a pre-existing LTC. Thus, we were unable to evaluate whether condition-tailored interventions were implemented in practice and how these may have influenced the outcomes. Our analysis did not consider different attributes of MLTCs, such as the sequence of the LTC diagnoses nor the time distance between the diagnoses. Future studies focusing specifically on MLTC attributes are warranted to provide a more nuanced understanding how different MLTC patterns may be associated with NHSTT treatment outcomes. Another limitation of the study is that the definition of working age (18–64) may not fully capture the employment experience of younger individuals in training or older adults who remain economically active beyond statutory pension age. Additionally, some patients may have sought therapeutic support outside the NHSTT settings (eg, private), which may have affected our observed estimates. Our study sample was restricted to patients attending NHSTT services within South London, a socioeconomically diverse and multiethnic region, and the findings may not generalise to patients from other geographical areas of the UK.

Our findings offer novel insights into potential variability for NHSTT treatment outcomes in working-age adults with physical LTCs. The findings highlight specific aspects in need of improvement. The apparent suboptimal likelihood of achieving ‘recovery’ and ‘reliable improvement’ observed in the study population reiterates the need for specialised training for therapists, and patient-centred therapeutic support that accounts for the complex interplay between mental and physical health. The findings for likelihood of achieving functional improvement underscore the necessity of a holistic approach to NHSTT service evaluation, which extends beyond symptoms severity to also monitor patients functioning in their daily lives. For policymakers, the results reinforce the critical importance of continuous service-level improvements to facilitate engagement with the treatment through adaptive use of current services for those with co-occurring mental and physical LTCs. For researchers, the findings underscore the need for investigations into the dynamic interplay of processes impacting poor treatment outcomes, to inform timing and targets for tailored interventions to reduce the burden of mental health in LTCs. Overall, these implications point to a pressing need to refine the NHSTT services and research agenda to meet the changing demands of the growing population living with burdensome mental and physical MLTC.

## Supplementary material

10.1136/bmjment-2025-301632 online supplemental file 1

## Data Availability

Data may be obtained from a third party and are not publicly available.

## References

[1] Clark DM (2011). Implementing NICE guidelines for the psychological treatment of depression and anxiety disorders: the IAPT experience. Int Rev Psychiatry.

[2] NHS Digital (2021). Psychological therapies: reports on the use of IAPT services, England.

[3] Seaton N, Moss-Morris R, Norton S (2022). Mental health outcomes in patients with a long-term condition: analysis of an Improving Access to Psychological Therapies service. BJPsych Open.

[4] Department of Health (2011). No health without mental health.

[5] Delgadillo J, Dawson A, Gilbody S (2017). Impact of long-term medical conditions on the outcomes of psychological therapy for depression and anxiety. Br J Psychiatry.

[6] Gruber J, Lordan G, Pilling S (2022). The impact of mental health support for the chronically ill on hospital utilisation: Evidence from the UK. Soc Sci Med.

[7] NHS Digital (2022). Psychological therapies, annual report on the use of IAPT services, 2021–22 England 2022.

[8] Cassell A, Edwards D, Harshfield A (2018). The epidemiology of multimorbidity in primary care: a retrospective cohort study. Br J Gen Pract.

[9] Kingston A, Robinson L, Booth H (2018). Projections of multi-morbidity in the older population in England to 2035: estimates from the Population Ageing and Care Simulation (PACSim) model. Age Ageing.

[10] Ronaldson A, Arias de la Torre J, Prina M (2021). Associations between physical multimorbidity patterns and common mental health disorders in middle-aged adults: A prospective analysis using data from the UK Biobank. *Lancet Reg Health Eur*.

[11] Glynn LG, Valderas JM, Healy P (2011). The prevalence of multimorbidity in primary care and its effect on health care utilization and cost. Fam Pract.

[12] Saunders R, Cape J, Leibowitz J (2020). Improvement in IAPT outcomes over time: are they driven by changes in clinical practice?. *Cogn Behav Therap*.

[13] Barnett K, Mercer SW, Norbury M (2012). Epidemiology of multimorbidity and implications for health care, research, and medical education: a cross-sectional study. Lancet.

[14] Ho I-S, Azcoaga-Lorenzo A, Akbari A (2021). Examining variation in the measurement of multimorbidity in research: a systematic review of 566 studies. Lancet Public Health.

[15] Arias-de la Torre J, Ronaldson A, Prina M (2021). Depressive symptoms during early adulthood and the development of physical multimorbidity in the UK: an observational cohort study. *Lancet Healthy Longev*.

[16] NICE (2021). Common mental health disorders: identification and pathways to care. (no. clinical guideline 123).

[17] Kroenke K, Spitzer RL, Williams JB (2001). The PHQ-9: validity of a brief depression severity measure. J Gen Intern Med.

[18] Spitzer RL, Kroenke K, Williams JBW (2006). A brief measure for assessing generalized anxiety disorder: the GAD-7. Arch Intern Med.

[19] Azur MJ, Stuart EA, Frangakis C (2011). Multiple imputation by chained equations: what is it and how does it work?. Int J Methods Psychiatr Res.

[20] Arundell L-LC, Saunders R, Buckman JEJ (2024). Differences in psychological treatment outcomes by ethnicity and gender: an analysis of individual patient data. Soc Psychiatry Psychiatr Epidemiol.

[21] Griffiths CA, Griffiths LJ (2015). Recovery and reliable change rates for patients scoring severe on depression, anxiety or impaired functioning in a psychological therapies service: IAPT. Ment Health Rev J.

[22] Dregan A, Matcham F, Harber-Aschan L (2019). Common mental disorders within chronic inflammatory disorders: a primary care database prospective investigation. Ann Rheum Dis.

[23] Harwood H, Rhead R, Chui Z (2023). Variations by ethnicity in referral and treatment pathways for IAPT service users in South London. Psychol Med.

