# Supplementary material for: Physical long-term conditions and the effectiveness of England’s NHS Talking Therapies programme for working-age adults: findings from a South London borough
Source: BMJ Ment Health. 2025 May 19;28(1):e301632. doi: 10.1136/bmjment-2025-301632 (PMC12090860; doi:10.1136/bmjment-2025-301632)

| Table S1. List of physical long-term conditions from Lambeth DataNet | |
| --- | --- |
| **Condition** | **Definition** |
| Asthma | Ever recorded |
| Atrial fibrillation | Ever recorded |
| Blindness | Ever recorded |
| Bronchiectasis | Ever recorded |
| Cancer | Recorded in previous 5 years |
| CHD | Ever recorded |
| CKD | Ever recorded |
| Chronic sinusitis | Ever recorded |
| COPD | Ever recorded |
| Dementia | Ever recorded |
| Diabetes | Ever recorded |
| Diverticular disease | Ever recorded |
| Epilepsy | Ever recorded |
| Glaucoma | Ever recorded |
| Hearing problems | Ever recorded |
| Heart failure | Ever recorded |
| HIV | Ever recorded |
| Hypertension | Ever recorded |
| IBS | Ever recorded |
| IBD | Ever recorded |
| Liver conditions | Ever recorded |
| MS | Ever recorded |
| Osteoporosis | Ever recorded |
| Parkinson’s disease | Ever recorded |
| Peptic ulcer | Ever recorded |
| PAD | Ever recorded |
| Prostate conditions | Ever recorded |
| Psoriasis/eczema | Ever recorded |
| Rheumatoid arthritis | Ever recorded |
| Stroke | Ever recorded |
| Thyroid problems | Ever recorded |

CHD=coronary heart disease; CKD=chronic kidney disease; COPD=chronic obstructive pulmonary disorder; HIV=human immunodeficiency virus; IBS=irritable bowel syndrome; IBD=inflammatory bowel disorder; MS=multiple sclerosis; PAD=peripheral arterial disease

Patients of working age (18-64y) who entered IAPT treatment from 2008 to March 2021

n=47,097

Included in analyses

n=35,814

Exclusions:

n= 7284: only one treatment session

n=3999: not clinical caseness

Figure S1. Patient flow diagram

| Table S2. Derived ethnicity variable from IAPT data | |  |
| --- | --- | --- |
| Ethnicity | Ethnic group | Ethnic subgroup |
| White  (N=23,122, 64.6%) | White | White British, White Irish, White Other |
| Black Caribbean  (N=3725, 10.4%) | Black/Black British | Black Caribbean |
| Black African  (N=1552, 4.3%) | Black/Black British | Other African, Somali, Nigerian, Sudanese, Angolan, Eritrean, Ethiopian, Ghanaian, Ugandan |
| Black Other  (N=870, 2.4%) | Black/Black British | Mixed Black, Other Black British, Other Black or Black unspecified |
| Asian  (N=1653, 4.6%) | Asian or Asian British | Indian, Pakistani, Bangladeshi, Mixed Asian, Punjabi, East African Asian, Sri Lankan, Tamil, Sinhalese, British Asian, Caribbean Asian, Other Asian or Asian unspecified, Vietnamese, Japanese, Filipino, Malaysian |
| Mixed ethnicity  (N=2475, 6.9%) | Mixed | White and Black Caribbean, White and Black African, White and Asian, Black and Asian, Black and Chinese, Black and White, Chinese and White, Asian and Chinese, Other mixed or mixed unspecified |
| Other ethnic group  (N=1347, 3.8%) | Other ethnic group | Chinese, Arab/Middle Eastern (Algerian, Middle Eastern, Arab, Iranian, Iraqi), Any Other Group, Columbian, Ecuadorian, Other Latin American |
| Not stated/unknown  (N=1070, 3.0%) |  |  |

Table S3. Fully-adjusted models for the association between specific LTCs with recovery and reliable improvement outcomes

|  | Recovery (n=6617) | | Reliable Improvement (n=6433) | |
| --- | --- | --- | --- | --- |
|  | *HR (95% CI)* | *p value* | *HR (95% CI)* | *p value* |
| Asthma (n=3257, 49.2%) | 0.97 (0.89 to 1.06) | 0.545 | 1.02 (0.94 to 1.11) | 0.589 |
| Atrial fibrillation (n=82, 1.2%) | 1.08 (0.71 to 1.65) | 0.712 | **1.44 (1.03 to 2.01)** | **0.034** |
| Blindness (n=145, 2.2%) | 1.06 (0.81 to 1.40) | 0.657 | 1.24 (0.96 to 1.60) | 0.102 |
| Bronchiectasis (n=30, 0.5%) | 1.16 (0.72 to 1.87) | 0.533 | 1.33 (0.88 to 2.01) | 0.179 |
| CHD (n=137, 2.1%) | 0.95 (0.68 to 1.32) | 0.746 | 1.07 (0.80 to 1.44) | 0.648 |
| CKD (n=171, 2.6%) | **1.47 (1.11 to 1.96)** | **0.008** | **1.43 (1.06 to 1.92)** | **0.019** |
| Chronic sinusitis (n=449, 6.8%) | 1.03 (0.87 to 1.22) | 0.319 | 1.08 (0.92 to 1.27) | 0.349 |
| COPD (n=114, 1.7%) | **0.62 (0.41 to 0.93)** | **0.021** | 0.86 (0.62 to 1.18) | 0.352 |
| Dementia (n=29, 0.4%) | 1.04 (0.54 to 1.98) | 0.913 | 1.09 (0.62 to 1.94) | 0.759 |
| Diabetes (n=1039, 15.7%) | **0.81 (0.71 to 0.92)** | **0.001** | 0.89 (0.79 to 1.00) | 0.060 |
| Diverticular disease (n=142, 2.1%) | 0.96 (0.65 to 1.43) | 0.862 | 1.10 (0.79 to 1.54) | 0.571 |
| Epilepsy (n=300, 4.5%) | 0.99 (0.80 to 1.21) | 0.902 | 1.03 (0.85 to 1.25) | 0.756 |
| Glaucoma (n=107, 1.6%) | 1.10 (0.78 to 1.55) | 0.582 | 1.20 (0.92 to 1.58) | 0.202 |
| Hearing problems (n=933, 14.1%) | 1.00 (0.89 to 1.12) | 0.956 | 0.96 (0.86 to 1.09) | 0.563 |
| Heart failure (n=48, 0.7%) | 1.30 (0.87 o 1.94) | 0.203 | 0.94 (0.58 to 1.55) | 0.823 |
| HIV (n=246, 3.7%) | 1.18 (0.96 to 1.44) | 0.107 | **1.26 (1.03 to 1.53)** | **0.021** |
| Hypertension (n=1375, 20.8%) | 0.90 (0.79 to 1.01) | 0.075 | 0.99 (0.88 to 1.11) | 0.854 |
| IBS (n=1587, 24.0%) | **0.88 (0.80 to 0.96)** | **0.006** | **0.83 (0.75 to 0.91)** | **<0.001** |
| IBD (n=163, 2.5%) | 0.86 (0.65 to 1.13) | 0.283 | 0.85 (0.64 to 1.11) | 0.236 |
| Liver conditions (n=188, 2.8%) | 0.98 (0.74 to 1.31) | 0.922 | 1.07 (0.64 to 1.78) | 0.795 |
| MS (n=46, 0.7%) | 1.23 (0.78 to 1.95) | 0.365 | 1.00 (0.71 to 1.42) | 0.989 |
| Osteoporosis (n=104, 1.6%) | 0.80 (0.54 to 1.20) | 0.293 | 0.73 (0.50 to 1.07) | 0.111 |
| Parkinson’s disease (n=3, 0.05%) | 1.38 (0.21 to 9.04) | 0.738 | 1.33 (0.21 to 8.37) | 0.758 |
| Peptic ulcer (n=136, 2.1%) | 0.95 (0.69 to 1.32) | 0.776 | 1.01 (0.73 to 1.38) | 0.971 |
| PAD (n=73, 1.1%) | 0.76 (0.47 to 1.24) | 0.276 | 1.06 (0.69 to 1.62) | 0.793 |
| Prostate conditions (n=105, 1.6%) | 0.80 (0.56 to 1.13) | 0.208 | 0.87 (0.62 to 1.22) | 0.431 |
| Psoriasis/eczema (n=3946, 59.6%) | **1.15 (1.05 to 1.25)** | **0.002** | 1.02 (0.94 to 1.11) | 0.573 |
| Rheumatoid arthritis (n=283, 4.3%) | 0.89 (0.72 to 1.10) | 0.278 | 0.97 (0.80 to 1.18) | 0.805 |
| Stroke (n=159, 2.4%) | 0.97 (0.69 to 1.37) | 0.884 | 1.07 (0.78 to 1.46) | 0.665 |
| Thyroid problems (n=676, 10.2%) | 1.04 (0.90 to 1.20) | 0.591 | 1.03 (0.89 to 1.19) | 0.717 |
| Cancer (n=71, 1.1%) | 0.80 (0.50 to 1.27) | 0.346 | 0.67 (0.41 to 1.09) | 0.104 |

Analyses adjusted for the following covariates: age, gender, ethnicity, employment status, index of multiple deprivation, baseline symptoms severity.

| Table S4. Sample characteristics for those included and those excluded from the study sample | | | |
| --- | --- | --- | --- |
|  | Analytical sample  (n=35,814) | Excluded sample  (n=11,283) |  |
|  | *M±SD or N(%)* | *M±SD or N(%)* |  |
| Age |  |  | **<0.001** |
| *18-24y* | 7758 (21.7) | 2148 (19.0) |  |
| *25-35y* | 14,636 (40.9) | 4586 (40.6) |  |
| *36-45y* | 7022 (19.6) | 2438 (21.6) |  |
| *46-55y* | 4593 (12.8) | 149 (13.3) |  |
| *56-65y* | 1805 (5.0) | 612 (5.4) |  |
| Female | 23,641 (66.0) | 7055 (62.5) | **<0.001** |
| Ethnicity |  |  | 0.674 |
| *White* | 23,122 (64.6) | 7281 (64.5) |  |
| *Black Caribbean* | 3725 (10.4) | 1216 (10.8) |  |
| *Black African* | 1552 (4.3) | 501 (4.4) |  |
| *Black Other* | 870 (2.4) | 273 (2.4) |  |
| *Asian* | 1653 (4.6) | 517 (4.6) |  |
| *Mixed ethnicity* | 2475 (6.9) | 722 (6.4) |  |
| *Other ethnicity* | 1347 (3.8) | 428 (3.8) |  |
| *Not stated* | 1070 (3.0) | 345 (3.1) |  |
| Employment status |  |  | **<0.001** |
| *Employed* | 24,085 (68.2) | 7664 (69.1) |  |
| *Unemployed* | 7857 (22.3) | 2409 (21.7) |  |
| *Full-time student* | 1885 (5.3) | 483 (4.3) |  |
| *Retired* | 185 (0.5) | 63 (0.6) |  |
| *Homemaker/carer* | 605 (1.7) | 196 (1.8) |  |
| *Long-term sick/disabled* | 670 (1.9) | 277 (2.5) |  |
| IMD quintile |  |  | 0.277 |
| *1 (least deprived)* | 7119 (20.5) | 2239 (20.4) |  |
| *2* | 6984 (20.1) | 2136 (19.5) |  |
| *3* | 6956 (20.0) | 2208 (20.1) |  |
| *4* | 6774 (19.5) | 2227 (20.3) |  |
| *5 (most deprived)* | 6951 (20.0) | 2151 (19.6) |  |
| Physical multimorbidity | 6617 (18.5) | 2086 (18.5) | 0.977 |
| Number physical diseases | 0.76±0.97 | 0.75±0.98 | 0.372 |
| Number of IAPT sessions | 9.11±6.28 | 3.41±4.35 | **<0.001** |
| Session DNA rate | 0.46±0.88 | 0.12±0.48 | **<0.001** |
| Episode duration (weeks) | 84.50±96.15 | 24.16±58.38 | **<0.001** |
| IAPT referral source |  |  | **<0.001** |
| *GP* | 6941 (19.4) | 2125 (18.8) |  |
| *Self-referral* | 25,944 (72.4) | 8047 (71.3) |  |
| *Other* | 2929 (8.2) | 1111 (9.8) |  |
| IAPT waiting time (days) | 17.82±28.91 | 17.78±28.05 | **0.003** |
| IAPT intervention |  |  | **<0.001** |
| *Step 2* | 12,681 (36.0) | 1747 (15.7) |  |
| *Step 3* | 13,953 (39.6) | 1622 (14.5) |  |
| *Other* | 8627 (24.4) | 7794 (69.8) |  |
| Total number of episodes | 1.32±0.69 | 1.29±0.66 | **0.002** |
| Baseline PHQ-9 | 15.58±5.60 | 11.21±7.40 | **<0.001** |
| Baseline GAD-7 | 14.24±4.31 | 9.81±6.25 | **<0.001** |
| Baseline WSAS | 17.51±8.04 | 11.88±8.27 | **<0.001** |
| Recovery | 13,604 (38.0) | - | **-** |
| Reliable improvement | 13,043 (36.4) | 280 (2.5) | **<0.001** |
| WSAS improvement | 2889 (8.1) | 497 (4.4) | **<0.001** |
| DNA=Did not attend; GAD-7=General Anxiety Disorder-7; GP=general practitioner; IMD=Index of multiple deprivation; LTC=Long-term conditions; M=mean; NHSTT=NHS Talking Therapies; PHQ-9=Patient Health Questionnaire-9; SD=standard deviation; RTT=Referral to treatment time. Covariates=age, gender, ethnicity, employment status, index of multiple deprivation, baseline symptoms severity.  *Other referral sources include general hospital/psychiatric services, community services, other.  **Step 2: low intensity interventions, e.g. guided self-help, behavioural activation, psychoeducation, and computerised CBT; Step 3: high intensity interventions, e.g. face-to-face CBT, interpersonal therapy, behavioural activation, counselling, psychodynamic therapy; Other: triage and therapies where the intensity was not recorded. | | | |


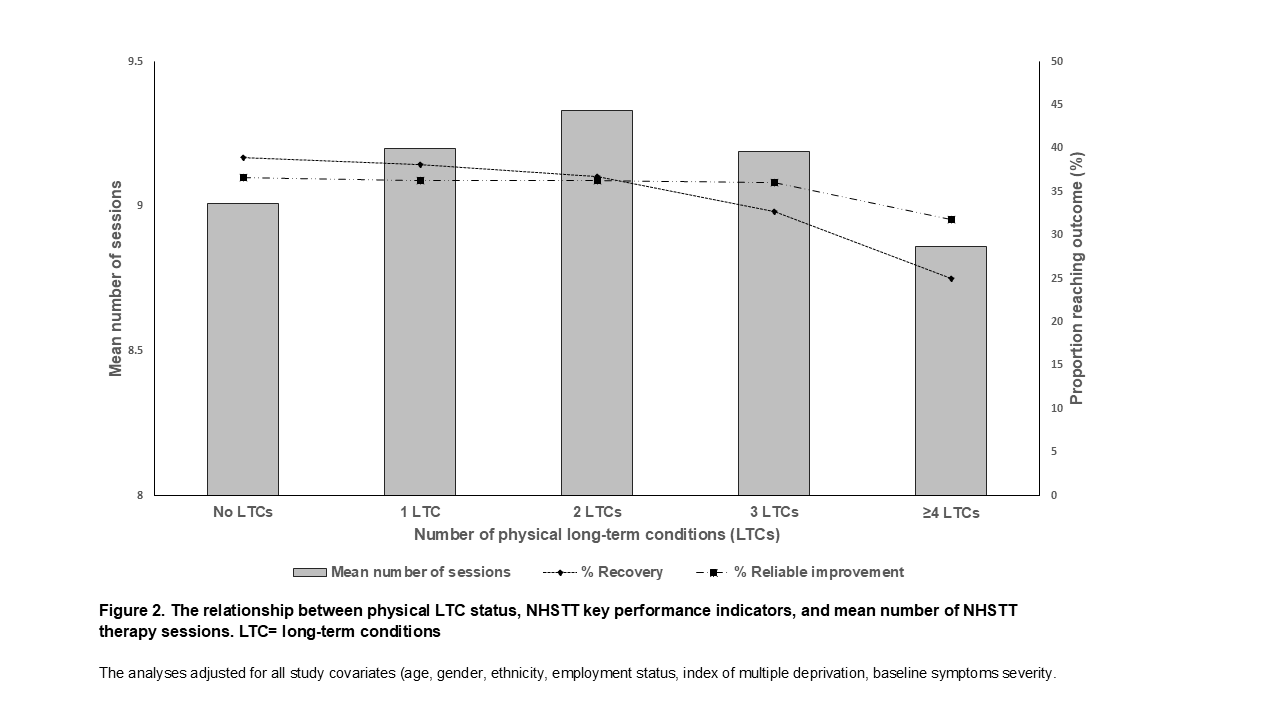


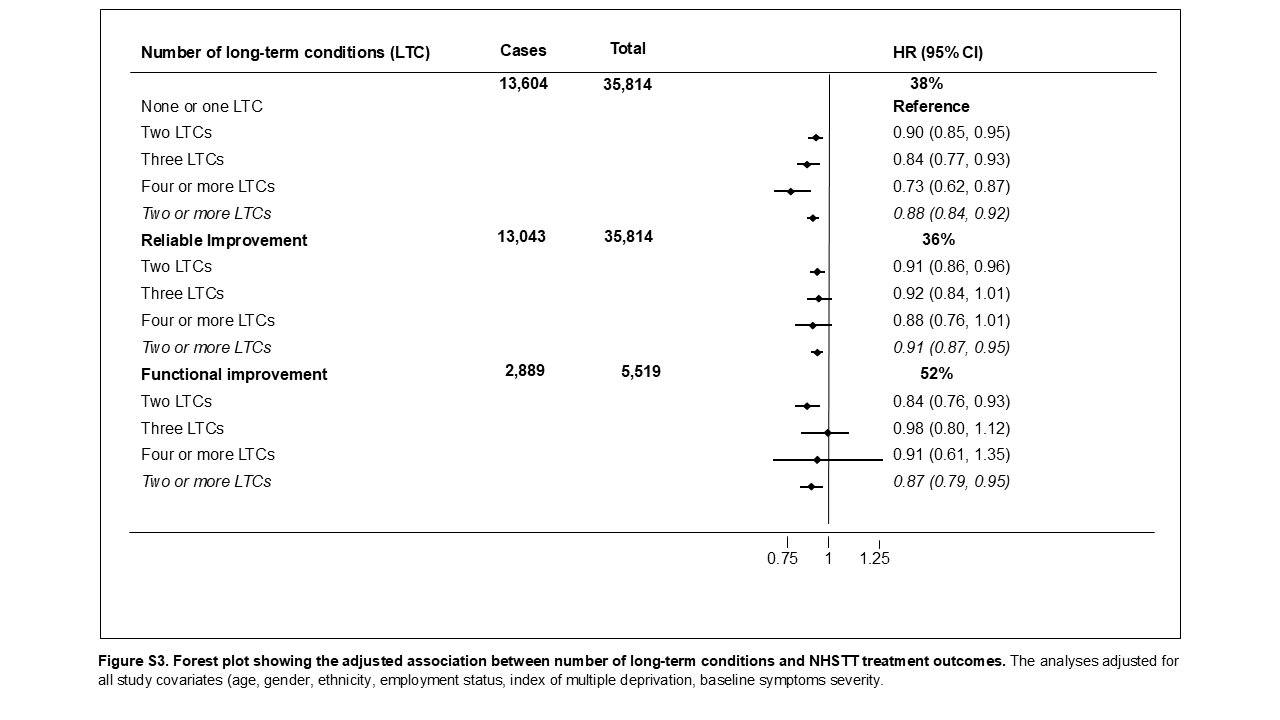

Supplement: online supplemental file 1 [file bmjment-28-1-s001.docx]
